# (Butan-2-ol-κ*O*)[2-({(ethyl­sulfan­yl)[2-(2-oxidobenzyl­idene-κ*O*)hydrazinyl­idene-κ*N* ^2^]meth­yl}imino­meth­yl)phenolato-κ*O*]dioxidouranium(VI)

**DOI:** 10.1107/S160053681200431X

**Published:** 2012-02-10

**Authors:** Reza Takjoo, Atefeh Najafi, Seik Weng Ng, Edward R. T. Tiekink

**Affiliations:** a Department of Chemistry, School of Sciences, Ferdowsi University of Mashhad, 91775-1436 Mashhad, Iran; b Department of Chemistry, University of Malaya, 50603 Kuala Lumpur, Malaysia; c Chemistry Department, Faculty of Science, King Abdulaziz University, PO Box 80203 Jeddah, Saudi Arabia

## Abstract

The U atom in the title complex, [U(C_17_H_15_N_3_O_2_S)O_2_(C_4_H_10_O)], exists within a distorted penta­gonal–bipyramidal geometry where the oxide O atoms occupy axial positions [O—U—O = 179.61 (18)°] and the penta­gonal plane is defined by the N_2_O_2_ atoms of the tetra­dentate Schiff base ligand and the O atom of the butan-2-ol mol­ecule. In the crystal, centrosymmetric aggregates are formed *via* pairs of hy­droxy–phenoxide O—H⋯O hydrogen bonds. The azomethine C=N atoms, the ethyl­thiolyl group and the butyl group of the butan-2-ol mol­ecule are disordered over two positions in a 0.668 (3):0.332 (3) ratio.

## Related literature

For background to uranyl Schiff base complexes, see: Şahin *et al.* (2010 ▶); Özdemir *et al.* (2011 ▶). For a related structure, see: Takjoo *et al.* (2012 ▶).
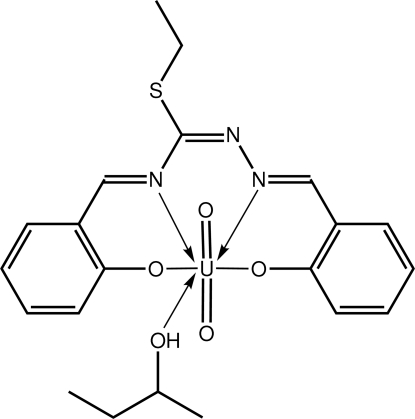


## Experimental

### 

#### Crystal data

[U(C_17_H_15_N_3_O_2_S)O_2_(C_4_H_10_O)]
*M* *_r_* = 669.53
Monoclinic, 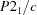

*a* = 11.3803 (2) Å
*b* = 14.3999 (3) Å
*c* = 14.0264 (4) Å
β = 97.326 (2)°
*V* = 2279.81 (9) Å^3^
*Z* = 4
Mo *K*α radiation
μ = 7.25 mm^−1^
*T* = 100 K
0.25 × 0.10 × 0.05 mm

#### Data collection

Agilent SuperNova Dual diffractometer with an Atlas detector
Absorption correction: multi-scan (*CrysAlis PRO*; Agilent, 2010 ▶) *T*_min_ = 0.265, *T*_max_ = 0.713
15878 measured reflections
5262 independent reflections
4493 reflections with *I* > 2σ(*I*)
*R*_int_ = 0.040

#### Refinement

*R*[*F* ^2^ > 2σ(*F* ^2^)] = 0.038
*wR*(*F* ^2^) = 0.078
*S* = 1.16
5262 reflections
302 parameters
8 restraints
H atoms treated by a mixture of independent and constrained refinement
Δρ_max_ = 1.16 e Å^−3^
Δρ_min_ = −0.81 e Å^−3^

### 

Data collection: *CrysAlis PRO* (Agilent, 2010 ▶); cell refinement: *CrysAlis PRO*; data reduction: *CrysAlis PRO*; program(s) used to solve structure: *SHELXS97* (Sheldrick, 2008 ▶); program(s) used to refine structure: *SHELXL97* (Sheldrick, 2008 ▶); molecular graphics: *X-SEED* (Barbour, 2001 ▶) and *DIAMOND* (Brandenburg, 2006 ▶); software used to prepare material for publication: *publCIF* (Westrip, 2010 ▶).

## Supplementary Material

Crystal structure: contains datablock(s) global, I. DOI: 10.1107/S160053681200431X/hb6617sup1.cif

Structure factors: contains datablock(s) I. DOI: 10.1107/S160053681200431X/hb6617Isup2.hkl

Additional supplementary materials:  crystallographic information; 3D view; checkCIF report

## Figures and Tables

**Table 1:** Selected bond lengths (Å)

U—O1	2.291 (4)
U—O2	2.229 (4)
U—O3	1.779 (4)
U—O4	1.776 (4)
U—O5	2.415 (4)
U—N1	2.562 (5)
U—N3	2.579 (5)

**Table 2:** Hydrogen-bond geometry (Å, °)

*D*—H⋯*A*	*D*—H	H⋯*A*	*D*⋯*A*	*D*—H⋯*A*
O5—H1o⋯O1^i^	0.84 (1)	1.83 (2)	2.648 (6)	166 (7)

Symmetry code: (i) 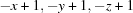.

**Fig. 1.**
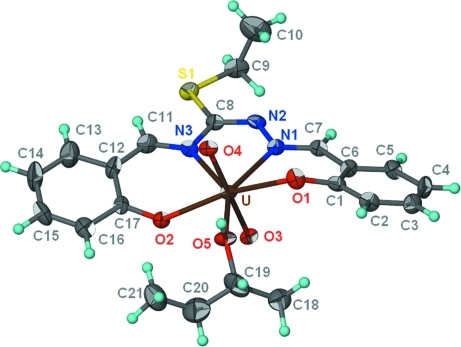
The molecular structure of (I) showing displacement ellipsoids at the 70% probability level. Only the major component of the disordered residues is shown.

**Fig. 2.**
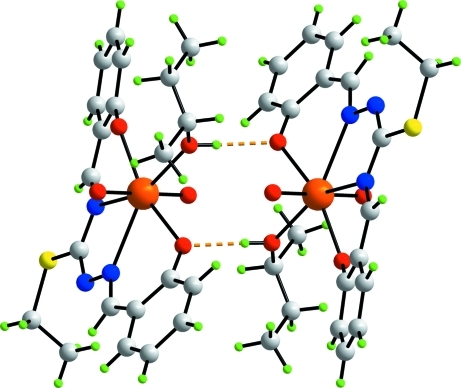
A view of the centrosymmetric aggregate in (I). The O—H···O hydrogen bonds are shown as dashed lines.

**Fig. 3.**
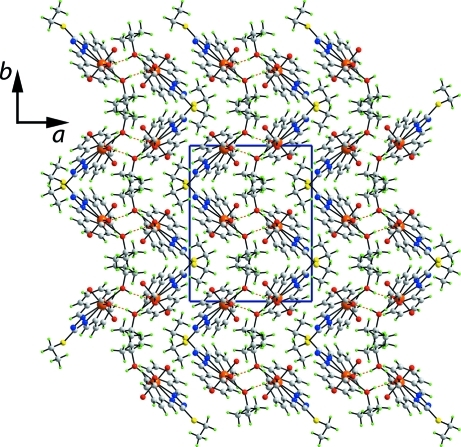
A view in projection down the c axis of the unit-cell contents of (I).

## supplementary crystallographic information

### Comment

Tetradentate ligands with N_2_O_2_ donor sets and their metal complexes attract attention as they provide synthetic models for the metal-containing sites in metallo-proteins and metallo-enzymes, and display extensive catalytic and bioactive applications. In this connection, recent studies of uranyl Schiff base complexes (Şahin *et al.*, 2010; Özdemir *et al.*, 2011) motivated the synthesis of the title complex, (I), in continuation of related studies (Takjoo *et al.*, 2012).

The U atom in (I), Fig. 1, exists within a distorted pentagonal bipyramidal geometry with the axial positions occupied by the oxido-O atoms, O3—U—O4 = 179.61 (18)°. The pentagonal plane is defined by the N_2_O_2_ atoms, derived from the tetradentate Schiff base ligand, and the O atom of the butan-2-ol molecule, Table 1. The Schiff base ligand is somewhat twisted with the dihedral angle between the terminal benzene rings being 31.7 (3)°.

In the crystal structure, centrosymmetric pairs of molecules are linked *via* O—H···O hydrogen bonds formed between the hydroxyl and O1-phenoxide atoms, Fig. 2 and Table 2. The dimeric aggregates stack into columns parallel to *c*, Fig. 3.

### Experimental

UO_2_(OAc)_2_.2H_2_O (0.42 g, 1.0 mmol) was added to a butanol solution (20 cm^3^) of salicylaldehyde mono-*S*-ethylisothiosemicarbazone hydrobromide (0.32 g, 1.0 mmol) and salicylaldehyde (0.12 g, 1.0 mmol). The red solution was heated under reflux for 1 h at 70 °C. Red prisms precipitated after four days, which were collected by filtration, washed with diethyl ether and dried in air. *M*.pt. 503 K. Yield: 25%.

### Refinement

Carbon-bound H-atoms were placed in calculated positions [C—H = 0.95 to 0.99 Å, *U*_iso_(H) = 1.2 to 1.5*U*_eq_(C)] and were included in the refinement in the riding model approximation. The hydroxyl H-atom was located in a difference Fourier map, and was refined with a distance restraint of O—H 0.84±0.01 Å and with *U*_iso_(H) = 1.5*U*_eq_(O).

The ethylthiolyl unit is disordered over two positions; the minor component refined to a site occupancy of 0.332 (3). The *U*_iso_ parameters of the atoms of the minor component were constrained to be equal to *U*_eq_ of the major component. Pairs of S—C and C—C distances were restrained to within 0.01 Å of each other. The azomethine C═N unit is also disordered; the positions and anisotropic displacement parameters of the primed atoms were set to those of the unprimed ones. The butan-2-ol molecule is also disordered over two positions with respect to the butyl portion only, and the occupancies were set to those of the ethylthiolyl unit. Pairs of C—O and C—C distances were restrained to within 0.01 Å.

The final difference Fourier map had a peak at 0.88 Å from C19 and a hole at 0.75 Å from U.

### Crystal data

**Table:**

[U(C_17_H_15_N_3_O_2_S)O_2_(C_4_H_10_O)]	*F*(000) = 1280
*M**_r_* = 669.53	*D*_x_ = 1.951 Mg m^−^^3^
Monoclinic, *P*2_1_/*c*	Mo *K*α radiation, λ = 0.71073 Å
Hall symbol: -P 2ybc	Cell parameters from 8576 reflections
*a* = 11.3803 (2) Å	θ = 2.2–27.5°
*b* = 14.3999 (3) Å	µ = 7.25 mm^−^^1^
*c* = 14.0264 (4) Å	*T* = 100 K
β = 97.326 (2)°	Prism, red
*V* = 2279.81 (9) Å^3^	0.25 × 0.10 × 0.05 mm
*Z* = 4	

### Data collection

**Table:**

Agilent SuperNova Dual diffractometer with an Atlas detector	5262 independent reflections
Radiation source: SuperNova (Mo) X-ray Source	4493 reflections with *I* > 2σ(*I*)
Mirror monochromator	*R*_int_ = 0.040
Detector resolution: 10.4041 pixels mm^-1^	θ_max_ = 27.6°, θ_min_ = 2.3°
ω scan	*h* = −14→14
Absorption correction: multi-scan (*CrysAlis PRO*; Agilent, 2010)	*k* = −18→18
*T*_min_ = 0.265, *T*_max_ = 0.713	*l* = −18→12
15878 measured reflections	

### Refinement

**Table:**

Refinement on *F*^2^	Primary atom site location: structure-invariant direct methods
Least-squares matrix: full	Secondary atom site location: difference Fourier map
*R*[*F*^2^ > 2σ(*F*^2^)] = 0.038	Hydrogen site location: inferred from neighbouring sites
*wR*(*F*^2^) = 0.078	H atoms treated by a mixture of independent and constrained refinement
*S* = 1.16	*w* = 1/[σ^2^(*F*_o_^2^) + (0.0199*P*)^2^ + 8.1201*P*] where *P* = (*F*_o_^2^ + 2*F*_c_^2^)/3
5262 reflections	(Δ/σ)_max_ = 0.001
302 parameters	Δρ_max_ = 1.16 e Å^−^^3^
8 restraints	Δρ_min_ = −0.81 e Å^−^^3^

### Fractional atomic coordinates and isotropic or equivalent isotropic displacement parameters (Å^2^)

**Table:**

	*x*	*y*	*z*	*U*_iso_*/*U*_eq_	Occ. (<1)
U	0.717677 (17)	0.479390 (14)	0.617403 (15)	0.01623 (7)	
S1	1.0356 (2)	0.23488 (16)	0.73678 (17)	0.0243 (6)	0.668 (3)
S1'	1.0688 (4)	0.2786 (3)	0.5310 (3)	0.024*	0.332 (3)
O1	0.6650 (3)	0.4824 (3)	0.4542 (3)	0.0239 (9)	
O2	0.7142 (4)	0.5085 (3)	0.7731 (3)	0.0228 (9)	
O3	0.8179 (3)	0.5740 (3)	0.6153 (3)	0.0190 (9)	
O4	0.6169 (3)	0.3854 (3)	0.6190 (3)	0.0195 (9)	
O5	0.5497 (3)	0.5832 (3)	0.6033 (3)	0.0237 (9)	
H1o	0.486 (3)	0.554 (4)	0.590 (5)	0.036*	
N1	0.8758 (4)	0.3968 (3)	0.5338 (3)	0.0186 (10)	
N2	0.9541 (4)	0.3318 (3)	0.5841 (4)	0.0194 (11)	0.668 (3)
C8'	0.9541 (4)	0.3318 (3)	0.5841 (4)	0.0194 (11)	0.33
N3	0.8570 (4)	0.3646 (3)	0.7175 (3)	0.0194 (10)	
C1	0.7233 (5)	0.5070 (4)	0.3817 (4)	0.0215 (13)	
C2	0.6682 (5)	0.5611 (4)	0.3058 (4)	0.0244 (13)	
H2	0.5910	0.5850	0.3088	0.029*	
C3	0.7248 (5)	0.5801 (4)	0.2268 (4)	0.0262 (14)	
H3	0.6865	0.6174	0.1763	0.031*	
C4	0.8382 (6)	0.5450 (4)	0.2201 (4)	0.0296 (15)	
H4	0.8764	0.5576	0.1651	0.036*	
C5	0.8933 (5)	0.4923 (4)	0.2941 (4)	0.0246 (14)	
H5	0.9699	0.4677	0.2894	0.030*	
C6	0.8393 (5)	0.4738 (4)	0.3765 (4)	0.0192 (12)	
C7	0.9043 (5)	0.4154 (4)	0.4491 (4)	0.0187 (12)	
H7	0.9751	0.3878	0.4331	0.022*	
C8	0.9416 (4)	0.3157 (4)	0.6716 (4)	0.0198 (12)	0.668 (3)
N2'	0.9416 (4)	0.3157 (4)	0.6716 (4)	0.0198 (12)	0.33
C9	1.1329 (10)	0.2017 (7)	0.6476 (9)	0.029 (3)	0.668 (3)
H9A	1.1528	0.2578	0.6121	0.035*	0.668 (3)
H9B	1.2076	0.1762	0.6813	0.035*	0.668 (3)
C9'	1.117 (2)	0.1895 (15)	0.621 (2)	0.029*	0.332 (3)
H9'A	1.1998	0.1722	0.6162	0.035*	0.332 (3)
H9'B	1.1135	0.2155	0.6862	0.035*	0.332 (3)
C10	1.0774 (11)	0.1312 (9)	0.5773 (9)	0.053 (4)	0.668 (3)
H10A	1.1324	0.1162	0.5312	0.080*	0.668 (3)
H10B	1.0040	0.1565	0.5429	0.080*	0.668 (3)
H10C	1.0595	0.0748	0.6118	0.080*	0.668 (3)
C10'	1.041 (3)	0.1044 (19)	0.609 (2)	0.053*	0.332 (3)
H10D	1.0712	0.0576	0.6562	0.080*	0.332 (3)
H10E	1.0417	0.0795	0.5438	0.080*	0.332 (3)
H10F	0.9591	0.1205	0.6179	0.080*	0.332 (3)
C11	0.8453 (5)	0.3368 (4)	0.8038 (4)	0.0254 (14)	
H11	0.8910	0.2843	0.8265	0.031*	
C12	0.7716 (5)	0.3761 (4)	0.8691 (4)	0.0259 (14)	
C13	0.7682 (6)	0.3306 (5)	0.9574 (5)	0.0398 (18)	
H13	0.8097	0.2737	0.9689	0.048*	
C14	0.7077 (7)	0.3653 (6)	1.0269 (5)	0.0422 (19)	
H14	0.7068	0.3329	1.0858	0.051*	
C15	0.6474 (6)	0.4485 (5)	1.0108 (5)	0.0320 (15)	
H15	0.6047	0.4731	1.0591	0.038*	
C16	0.6485 (5)	0.4960 (4)	0.9258 (5)	0.0277 (14)	
H16	0.6073	0.5534	0.9165	0.033*	
C17	0.7097 (5)	0.4610 (4)	0.8523 (4)	0.0198 (13)	
C18	0.6045 (6)	0.7293 (5)	0.5245 (5)	0.0350 (16)	
H18A	0.5949	0.7969	0.5244	0.053*	
H18B	0.5833	0.7054	0.4591	0.053*	
H18C	0.6872	0.7135	0.5471	0.053*	
C19	0.5234 (10)	0.6853 (7)	0.5916 (8)	0.044 (3)	0.668 (3)
H19	0.4405	0.6907	0.5585	0.053*	0.668 (3)
C19'	0.574 (2)	0.6858 (9)	0.6188 (10)	0.044*	0.332 (3)
H19'	0.6420	0.6942	0.6704	0.053*	0.332 (3)
C20	0.5226 (12)	0.7204 (9)	0.6886 (9)	0.051 (3)	0.668 (3)
H20A	0.6052	0.7169	0.7207	0.062*	0.668 (3)
H20B	0.5022	0.7872	0.6827	0.062*	0.668 (3)
C20'	0.468 (2)	0.725 (2)	0.650 (2)	0.051*	0.332 (3)
H20C	0.4803	0.7928	0.6609	0.062*	0.332 (3)
H20D	0.4010	0.7179	0.5981	0.062*	0.332 (3)
C21	0.4487 (16)	0.6807 (19)	0.7538 (10)	0.042 (4)	0.668 (3)
H21A	0.4618	0.7135	0.8154	0.063*	0.668 (3)
H21B	0.4686	0.6149	0.7639	0.063*	0.668 (3)
H21C	0.3654	0.6865	0.7265	0.063*	0.668 (3)
C21'	0.434 (4)	0.685 (4)	0.737 (3)	0.042*	0.332 (3)
H21D	0.3639	0.7177	0.7544	0.063*	0.332 (3)
H21E	0.4992	0.6910	0.7889	0.063*	0.332 (3)
H21F	0.4146	0.6193	0.7256	0.063*	0.332 (3)

### Atomic displacement parameters (Å^2^)

**Table:**

	*U*^11^	*U*^22^	*U*^33^	*U*^12^	*U*^13^	*U*^23^
U	0.01237 (10)	0.01495 (11)	0.02069 (11)	0.00117 (9)	−0.00049 (8)	0.00006 (10)
S1	0.0231 (11)	0.0219 (12)	0.0267 (13)	0.0079 (9)	−0.0014 (10)	0.0046 (10)
O1	0.0144 (19)	0.033 (2)	0.024 (2)	0.0056 (18)	−0.0006 (17)	0.0000 (19)
O2	0.026 (2)	0.020 (2)	0.022 (2)	−0.0008 (17)	0.0038 (18)	−0.0006 (17)
O3	0.0125 (18)	0.018 (2)	0.028 (2)	0.0010 (16)	0.0060 (17)	0.0025 (17)
O4	0.0133 (19)	0.018 (2)	0.028 (2)	0.0001 (16)	0.0037 (17)	−0.0015 (17)
O5	0.018 (2)	0.016 (2)	0.037 (3)	0.0032 (17)	0.004 (2)	−0.0016 (19)
N1	0.016 (2)	0.019 (2)	0.019 (3)	0.002 (2)	−0.005 (2)	0.001 (2)
N2	0.018 (3)	0.016 (3)	0.023 (3)	0.006 (2)	−0.001 (2)	−0.003 (2)
C8'	0.018 (3)	0.016 (3)	0.023 (3)	0.006 (2)	−0.001 (2)	−0.003 (2)
N3	0.015 (2)	0.022 (3)	0.021 (3)	0.002 (2)	0.000 (2)	−0.002 (2)
C1	0.023 (3)	0.018 (3)	0.022 (3)	0.001 (2)	−0.001 (3)	−0.003 (2)
C2	0.018 (3)	0.027 (3)	0.025 (3)	0.007 (3)	−0.005 (3)	0.000 (3)
C3	0.026 (3)	0.028 (3)	0.024 (3)	0.007 (3)	−0.001 (3)	0.005 (3)
C4	0.038 (4)	0.034 (4)	0.016 (3)	−0.001 (3)	0.003 (3)	0.007 (3)
C5	0.023 (3)	0.030 (4)	0.021 (3)	0.000 (3)	0.002 (3)	−0.003 (3)
C6	0.015 (3)	0.022 (3)	0.019 (3)	0.003 (2)	−0.004 (2)	0.003 (3)
C7	0.012 (3)	0.021 (3)	0.024 (3)	0.002 (2)	0.003 (2)	−0.002 (2)
C8	0.016 (3)	0.019 (3)	0.024 (3)	0.001 (2)	0.002 (2)	−0.002 (2)
N2'	0.016 (3)	0.019 (3)	0.024 (3)	0.001 (2)	0.002 (2)	−0.002 (2)
C9	0.018 (6)	0.025 (6)	0.042 (8)	0.012 (4)	−0.004 (5)	0.002 (5)
C10	0.044 (8)	0.051 (9)	0.062 (9)	0.027 (7)	−0.002 (6)	−0.009 (7)
C11	0.020 (3)	0.026 (3)	0.029 (3)	0.008 (3)	−0.003 (3)	0.004 (3)
C12	0.023 (3)	0.032 (4)	0.023 (3)	0.001 (3)	0.004 (3)	0.010 (3)
C13	0.041 (4)	0.047 (5)	0.033 (4)	0.020 (4)	0.010 (3)	0.013 (3)
C14	0.045 (4)	0.061 (5)	0.022 (4)	0.012 (4)	0.009 (3)	0.014 (3)
C15	0.028 (3)	0.047 (4)	0.022 (3)	0.007 (3)	0.004 (3)	−0.009 (3)
C16	0.020 (3)	0.034 (4)	0.029 (3)	−0.001 (3)	0.003 (3)	−0.003 (3)
C17	0.017 (3)	0.026 (3)	0.016 (3)	−0.008 (2)	−0.001 (2)	0.000 (2)
C18	0.033 (4)	0.035 (4)	0.035 (4)	−0.002 (3)	−0.002 (3)	0.000 (3)
C19	0.038 (7)	0.049 (7)	0.050 (7)	0.005 (6)	0.020 (6)	−0.016 (6)
C20	0.051 (8)	0.046 (7)	0.057 (9)	−0.018 (6)	0.005 (6)	−0.002 (6)
C21	0.041 (8)	0.056 (8)	0.026 (7)	0.010 (6)	−0.008 (7)	−0.005 (7)

### Geometric parameters (Å, º)

**Table:**

U—O1	2.291 (4)	C10—H10B	0.9800
U—O2	2.229 (4)	C10—H10C	0.9800
U—O3	1.779 (4)	C10'—H10D	0.9800
U—O4	1.776 (4)	C10'—H10E	0.9800
U—O5	2.415 (4)	C10'—H10F	0.9800
U—N1	2.562 (5)	C11—C12	1.435 (9)
U—N3	2.579 (5)	C11—H11	0.9500
S1—C8	1.757 (5)	C12—C13	1.405 (9)
S1—C9	1.836 (12)	C12—C17	1.416 (8)
S1'—C9'	1.835 (15)	C13—C14	1.357 (10)
O1—C1	1.331 (7)	C13—H13	0.9500
O2—C17	1.311 (7)	C14—C15	1.385 (10)
O5—C19	1.506 (10)	C14—H14	0.9500
O5—C19'	1.513 (13)	C15—C16	1.376 (9)
O5—H1o	0.839 (10)	C15—H15	0.9500
N1—C7	1.299 (7)	C16—C17	1.409 (8)
N1—N2	1.417 (6)	C16—H16	0.9500
N2—C8	1.274 (7)	C18—C19	1.537 (11)
N3—C11	1.298 (8)	C18—C19'	1.544 (13)
N3—C8	1.412 (7)	C18—H18A	0.9800
C1—C2	1.402 (8)	C18—H18B	0.9800
C1—C6	1.414 (8)	C18—H18C	0.9800
C2—C3	1.378 (9)	C19—C20	1.453 (13)
C2—H2	0.9500	C19—H19	1.0000
C3—C4	1.399 (9)	C19'—C20'	1.455 (15)
C3—H3	0.9500	C19'—H19'	1.0000
C4—C5	1.371 (8)	C20—C21	1.438 (14)
C4—H4	0.9500	C20—H20A	0.9900
C5—C6	1.402 (8)	C20—H20B	0.9900
C5—H5	0.9500	C20'—C21'	1.439 (16)
C6—C7	1.449 (8)	C20'—H20C	0.9900
C7—H7	0.9500	C20'—H20D	0.9900
C9—C10	1.498 (13)	C21—H21A	0.9800
C9—H9A	0.9900	C21—H21B	0.9800
C9—H9B	0.9900	C21—H21C	0.9800
C9'—C10'	1.497 (15)	C21'—H21D	0.9800
C9'—H9'A	0.9900	C21'—H21E	0.9800
C9'—H9'B	0.9900	C21'—H21F	0.9800
C10—H10A	0.9800		
			
O4—U—O3	179.61 (18)	H10A—C10—H10B	109.5
O4—U—O2	92.23 (16)	C9—C10—H10C	109.5
O3—U—O2	87.92 (16)	H10A—C10—H10C	109.5
O4—U—O1	86.55 (17)	H10B—C10—H10C	109.5
O3—U—O1	93.18 (16)	C9'—C10'—H10D	109.5
O2—U—O1	160.07 (14)	C9'—C10'—H10E	109.5
O4—U—O5	88.12 (15)	H10D—C10'—H10E	109.5
O3—U—O5	91.55 (15)	C9'—C10'—H10F	109.5
O2—U—O5	81.44 (15)	H10D—C10'—H10F	109.5
O1—U—O5	78.64 (14)	H10E—C10'—H10F	109.5
O4—U—N1	98.29 (16)	N3—C11—C12	127.8 (5)
O3—U—N1	81.88 (16)	N3—C11—H11	116.1
O2—U—N1	129.55 (14)	C12—C11—H11	116.1
O1—U—N1	70.20 (14)	C13—C12—C17	118.7 (6)
O5—U—N1	147.64 (15)	C13—C12—C11	117.6 (6)
O4—U—N3	81.81 (16)	C17—C12—C11	123.5 (5)
O3—U—N3	98.57 (16)	C14—C13—C12	122.3 (7)
O2—U—N3	70.97 (15)	C14—C13—H13	118.8
O1—U—N3	128.32 (15)	C12—C13—H13	118.8
O5—U—N3	150.10 (15)	C13—C14—C15	119.2 (6)
N1—U—N3	62.11 (15)	C13—C14—H14	120.4
C8—S1—C9	101.4 (4)	C15—C14—H14	120.4
C1—O1—U	132.5 (4)	C16—C15—C14	120.8 (6)
C17—O2—U	137.7 (4)	C16—C15—H15	119.6
C19—O5—U	139.3 (5)	C14—C15—H15	119.6
C19'—O5—U	117.8 (9)	C15—C16—C17	121.1 (6)
C19—O5—H1o	108 (5)	C15—C16—H16	119.5
C19'—O5—H1o	131 (5)	C17—C16—H16	119.5
U—O5—H1o	111 (5)	O2—C17—C16	120.6 (5)
C7—N1—N2	112.1 (5)	O2—C17—C12	121.3 (5)
C7—N1—U	126.5 (4)	C16—C17—C12	118.0 (5)
N2—N1—U	120.9 (3)	C19—C18—H18A	109.5
C8—N2—N1	117.3 (4)	C19—C18—H18B	109.5
C11—N3—C8	115.4 (5)	H18A—C18—H18B	109.5
C11—N3—U	125.1 (4)	C19—C18—H18C	109.5
C8—N3—U	118.6 (3)	H18A—C18—H18C	109.5
O1—C1—C2	120.7 (5)	H18B—C18—H18C	109.5
O1—C1—C6	120.6 (5)	C20—C19—O5	105.2 (9)
C2—C1—C6	118.6 (6)	C20—C19—C18	120.4 (9)
C3—C2—C1	120.7 (6)	O5—C19—C18	110.0 (6)
C3—C2—H2	119.6	C20—C19—H19	106.8
C1—C2—H2	119.6	O5—C19—H19	106.8
C2—C3—C4	120.8 (6)	C18—C19—H19	106.8
C2—C3—H3	119.6	C20'—C19'—O5	106.5 (17)
C4—C3—H3	119.6	C20'—C19'—C18	113.0 (17)
C5—C4—C3	119.1 (6)	O5—C19'—C18	109.2 (8)
C5—C4—H4	120.5	C20'—C19'—H19'	109.3
C3—C4—H4	120.5	O5—C19'—H19'	109.3
C4—C5—C6	121.5 (6)	C18—C19'—H19'	109.3
C4—C5—H5	119.3	C21—C20—C19	122.1 (13)
C6—C5—H5	119.3	C21—C20—H20A	106.8
C5—C6—C1	119.3 (5)	C19—C20—H20A	106.8
C5—C6—C7	116.8 (5)	C21—C20—H20B	106.8
C1—C6—C7	123.8 (5)	C19—C20—H20B	106.8
N1—C7—C6	126.7 (5)	H20A—C20—H20B	106.7
N1—C7—H7	116.7	C21'—C20'—C19'	115 (3)
C6—C7—H7	116.7	C21'—C20'—H20C	108.6
N2—C8—N3	121.0 (4)	C19'—C20'—H20C	108.6
N2—C8—S1	119.1 (4)	C21'—C20'—H20D	108.6
N3—C8—S1	119.9 (4)	C19'—C20'—H20D	108.6
C10—C9—S1	112.6 (9)	H20C—C20'—H20D	107.5
C10—C9—H9A	109.1	C20—C21—H21A	109.5
S1—C9—H9A	109.1	C20—C21—H21B	109.5
C10—C9—H9B	109.1	H21A—C21—H21B	109.5
S1—C9—H9B	109.1	C20—C21—H21C	109.5
H9A—C9—H9B	107.8	H21A—C21—H21C	109.5
C10'—C9'—S1'	112.0 (17)	H21B—C21—H21C	109.5
C10'—C9'—H9'A	109.2	C20'—C21'—H21D	109.5
S1'—C9'—H9'A	109.2	C20'—C21'—H21E	109.5
C10'—C9'—H9'B	109.2	H21D—C21'—H21E	109.5
S1'—C9'—H9'B	109.2	C20'—C21'—H21F	109.5
H9'A—C9'—H9'B	107.9	H21D—C21'—H21F	109.5
C9—C10—H10A	109.5	H21E—C21'—H21F	109.5
C9—C10—H10B	109.5		
			
O4—U—O1—C1	−150.2 (5)	C2—C3—C4—C5	−0.9 (10)
O3—U—O1—C1	30.1 (5)	C3—C4—C5—C6	−0.8 (10)
O2—U—O1—C1	122.8 (5)	C4—C5—C6—C1	2.7 (9)
O5—U—O1—C1	121.0 (5)	C4—C5—C6—C7	178.5 (6)
N1—U—O1—C1	−50.1 (5)	O1—C1—C6—C5	172.9 (5)
N3—U—O1—C1	−73.5 (5)	C2—C1—C6—C5	−2.9 (8)
O4—U—O2—C17	37.7 (5)	O1—C1—C6—C7	−2.6 (9)
O3—U—O2—C17	−142.6 (5)	C2—C1—C6—C7	−178.4 (5)
O1—U—O2—C17	123.7 (6)	N2—N1—C7—C6	175.5 (5)
O5—U—O2—C17	125.5 (5)	U—N1—C7—C6	−12.5 (8)
N1—U—O2—C17	−65.0 (6)	C5—C6—C7—N1	173.2 (6)
N3—U—O2—C17	−42.8 (5)	C1—C6—C7—N1	−11.2 (9)
O4—U—O5—C19	−176.8 (7)	N1—N2—C8—N3	−3.1 (8)
O3—U—O5—C19	3.0 (7)	N1—N2—C8—S1	179.5 (4)
O2—U—O5—C19	90.7 (7)	C11—N3—C8—N2	174.3 (5)
O1—U—O5—C19	−89.9 (7)	U—N3—C8—N2	4.1 (7)
N1—U—O5—C19	−74.2 (8)	C11—N3—C8—S1	−8.3 (7)
N3—U—O5—C19	113.3 (7)	U—N3—C8—S1	−178.5 (2)
O4—U—O5—C19'	167.3 (6)	C9—S1—C8—N2	2.3 (6)
O3—U—O5—C19'	−12.9 (6)	C9—S1—C8—N3	−175.1 (5)
O2—U—O5—C19'	74.8 (6)	C8—S1—C9—C10	−80.6 (9)
O1—U—O5—C19'	−105.8 (6)	C8—N3—C11—C12	176.4 (6)
N1—U—O5—C19'	−90.1 (6)	U—N3—C11—C12	−14.1 (9)
N3—U—O5—C19'	97.4 (6)	N3—C11—C12—C13	176.4 (7)
O4—U—N1—C7	113.7 (5)	N3—C11—C12—C17	−8.4 (10)
O3—U—N1—C7	−66.0 (5)	C17—C12—C13—C14	0.0 (11)
O2—U—N1—C7	−146.5 (4)	C11—C12—C13—C14	175.5 (7)
O1—U—N1—C7	30.4 (4)	C12—C13—C14—C15	−0.2 (12)
O5—U—N1—C7	14.0 (6)	C13—C14—C15—C16	−0.2 (11)
N3—U—N1—C7	−170.2 (5)	C14—C15—C16—C17	0.8 (10)
O4—U—N1—N2	−75.1 (4)	U—O2—C17—C16	−145.9 (5)
O3—U—N1—N2	105.3 (4)	U—O2—C17—C12	37.9 (8)
O2—U—N1—N2	24.8 (4)	C15—C16—C17—O2	−177.3 (6)
O1—U—N1—N2	−158.4 (4)	C15—C16—C17—C12	−1.0 (9)
O5—U—N1—N2	−174.8 (3)	C13—C12—C17—O2	176.9 (6)
N3—U—N1—N2	1.0 (3)	C11—C12—C17—O2	1.7 (9)
C7—N1—N2—C8	173.0 (5)	C13—C12—C17—C16	0.6 (9)
U—N1—N2—C8	0.6 (6)	C11—C12—C17—C16	−174.6 (6)
O4—U—N3—C11	−67.7 (5)	C19'—O5—C19—C20	−58.1 (15)
O3—U—N3—C11	112.3 (5)	U—O5—C19—C20	−93.5 (9)
O2—U—N3—C11	27.6 (5)	C19'—O5—C19—C18	73.0 (11)
O1—U—N3—C11	−146.7 (4)	U—O5—C19—C18	37.6 (12)
O5—U—N3—C11	3.9 (6)	C19'—C18—C19—C20	49.7 (16)
N1—U—N3—C11	−171.6 (5)	C19'—C18—C19—O5	−72.9 (11)
O4—U—N3—C8	101.5 (4)	C19—O5—C19'—C20'	51.0 (19)
O3—U—N3—C8	−78.5 (4)	U—O5—C19'—C20'	−154.3 (14)
O2—U—N3—C8	−163.3 (4)	C19—O5—C19'—C18	−71.3 (11)
O1—U—N3—C8	22.5 (4)	U—O5—C19'—C18	83.4 (14)
O5—U—N3—C8	173.0 (3)	C19—C18—C19'—C20'	−47 (2)
N1—U—N3—C8	−2.5 (3)	C19—C18—C19'—O5	71.2 (11)
U—O1—C1—C2	−136.4 (5)	O5—C19—C20—C21	−54.6 (18)
U—O1—C1—C6	47.9 (8)	C18—C19—C20—C21	−179.5 (14)
O1—C1—C2—C3	−174.5 (6)	O5—C19'—C20'—C21'	58 (4)
C6—C1—C2—C3	1.3 (9)	C18—C19'—C20'—C21'	178 (3)
C1—C2—C3—C4	0.6 (10)		

### Hydrogen-bond geometry (Å, º)

**Table:**

*D*—H···*A*	*D*—H	H···*A*	*D*···*A*	*D*—H···*A*
O5—H1*o*···O1^i^	0.84 (1)	1.83 (2)	2.648 (6)	166 (7)

Symmetry code: (i) −*x*+1, −*y*+1, −*z*+1.

## References

[bb1] Agilent (2010). *CrysAlis PRO* Agilent Technologies, Yarnton, Oxfordshire, England.

[bb2] Barbour, L. J. (2001). *J. Supramol. Chem.* **1**, 189–191.

[bb3] Brandenburg, K. (2006). *DIAMOND* Crystal Impact GbR, Bonn, Germany.

[bb4] Özdemir, N., Şahin, M., Bal-Demirci, T. & Ülküseven, B. (2011). *Polyhedron*, **30**, 515–521.

[bb5] Şahin, M., Koca, A., Özdemir, N., Dinçer, M., Büyükgüngör, O., Bal-Demirci, T. & Ülküseven, B. (2010). *Dalton Trans.* **39**, 10228–10237. 10.1039/c0dt00265h 20922238

[bb6] Sheldrick, G. M. (2008). *Acta Cryst.* A**64**, 112–122. 10.1107/S0108767307043930 18156677

[bb7] Takjoo, R., Ng, S. W. & Tiekink, E. R. T. (2012). *Acta Cryst* E**68**, m244–m245. 10.1107/S1600536812003789 PMC3297215 22412405

[bb8] Westrip, S. P. (2010). *J. Appl. Cryst.* **43**, 920–925.

